# 

**Published:** 1893-08

**Authors:** 


### CONTENTS.

COMMUNICATIONS.

Little Things.......... 365
Elements of Success.......... 371
A Talk on Combination Fillings.......... 375
The Elevator—Its principle and Utility in Extraction.......... 377

PROCEEDINGS.

The Alabama Dental Association.......... 384

SELECTIONS.

A Chemical Test of the Antiquity of Bones.......... 403
The Relation of Suicide to Alcohol Consumption.......... 404
Cocaine in Surgery.......... 405

NOTICES.

Southern Dental Association.......... 406
National Association of Dental Examiners.......... 406
American Dental Association.......... 406

EDITORIAL.

Registration of Foreign Diplomas in England.......... 407

